# Does early childbearing affect utilization of antenatal care services and infant birth weight: Evidence from West and Central African Region

**DOI:** 10.7189/jogh.11.13003

**Published:** 2021-08-10

**Authors:** Vera Sagalova, Anne-Sophie Le Dain, Till Bärnighausen, Noel Marie Zagre, Sebastian Vollmer

**Affiliations:** 1 Heidelberg Institute of Global Health, University of Heidelberg, Heidelberg, Germany; 2 United Nations Children’s Fund (UNICEF) West and Central Africa Regional Office, Dakar, Senegal; 3 UNICEF Area Representative for Gabon and São Tomé and Príncipe and to the ECCAS, Libreville, Gabon; 4 Department of Economics and Centre for Modern Indian Studies, University of Göttingen, Göttingen, Germany

## Abstract

**Background:**

Adequate antenatal care (ANC) utilization is recognized as one of the important drivers of safe childbirth and positive birth outcomes. The usage of ANC services fluctuates with various personal, socio-economic, and cultural characteristics and in resource-poor settings, adolescent mothers are at a particularly high risk of insufficient ANC utilization.

**Objectives:**

This paper investigates whether the usage of ANC services and institutional delivery as well as newborn birth weight differ systematically between adolescent and adult mothers in West and Central Africa. Moreover, we explore to what extent differences in birth weight are explained by ANC usage, adolescence, and select socio-economic characteristics of the mother.

**Methods:**

We pooled cross-sectional data from all Demographic and Health Surveys (DHS) and Multi Indicator Cluster Surveys (MICS) conducted in countries in West and Central Africa region between 1986 and 2017 to estimate measures of ANC usage and qualified delivery assistance (along with a combined measure of “adequate maternal healthcare” aggregating these two factors) and newborn birth weight by maternal age group. We estimated various regression models to analyze a) the association between adolescence and adequate prenatal and maternal health care controlling for select socio-economic maternal characteristics as well as the local environment and b) between adolescence, adequate maternal health care, and newborn birth weight outcomes, also controlling for maternal characteristics and the local environment. All regressions were linear probability models for binary outcomes and simple linear models for continuous outcomes.

**Results:**

Adequate maternal health care provision was lowest among adolescent mothers: 23.0% among adolescents vs an average of 29.2% across all other age groups. Moreover, we found maternal education and wealth to be positively and significantly associated with receiving adequate maternal health care. Adolescent mothers had the highest risk of low infantile birth weight with 14.5% (95% confidence interval (CI) = 13.6%-15.5%), which is roughly 1.5-2 times higher than in older mothers. We found that adolescence is still strongly associated with low birth weight even when adequate maternal health care and various socio-economic factors as well as the local environment are controlled for.

**Conclusions:**

Our findings suggest that ANC supply in resource-poor settings should be particularly tailored to adolescent mothers’ needs and that further research is necessary to explore what individual maternal characteristics beyond socio-economic and physical (eg, BMI) factors drive the prevalence of low birth weight. Moreover, the currently used measures of maternal care quality are heavily dependent on pure quantitative measures (number of ANC visits). New indicators incorporating measures of factual quality and scope ought to be developed and incorporated into large routine household surveys such as DHS and MICS.

Antenatal care (ANC) is a concept that goes far beyond mere medical treatment of pregnant women; it is rather an umbrella term for a myriad of important health care functions such as health promotion and disease prevention, screening and diagnosis, information provision, dietary guidance, social, cultural, emotional and psychological support, and in some cases even a network to connect with that stretches far into motherhood [1].

Empirical studies have consistently shown that women who adhere to an adequate ANC plan have lower maternal and perinatal mortality and better pregnancy outcomes. Moreover, studies have shown a positive association between pregnancy outcomes and the number of ANC visits or the time of first ANC visit/gestational age [2,3].

Recommendations on the exact schedule and number of ANC visits differ across regions and time. At the beginning of 2020, the current WHO (World Health Organization) ANC model recommends a minimum of eight contacts with a qualified health provider throughout pregnancy, with the first contact taking place within the first 12 weeks of gestation. In the early 2000s, a so-called ‘focused’ ANC model (FANC, developed in the 1990s) has been promoted by the WHO in resource-poor settings. This model prescribes a minimum of four ‘goal-oriented’ ANC visits during a pregnancy [4]. However, empirical evidence suggests that adherence to this reduced model is associated with a higher risk of adverse pregnancy outcomes compared to the standard ANC schedule. [2,5] The reduced model was therefore dropped and is no longer recommended by the WHO. However, “ANC 4+” (proportion of pregnant women receiving four or more ANC visits) is widely used as a global benchmark indicator to assess the adequacy of antenatal care throughout empirical literature focusing on middle- and low-income settings [6].

Moreover, ANC utilization is shown to be positively correlated with institutional delivery and skilled birth attendance – another important determinant of safe childbirth. In addition to this, institutional delivery is also inextricably linked with socio-economic factors such as household wealth and education of the mother [7,8].

Empirical population-based studies confirm that in resource-poor settings and in particular in Sub-Saharan Africa, ANC usage is associated with both household wealth and maternal education [9].

As we have shown in previous work [10], adolescents in resource-poor settings experience “overlapping vulnerabilities”, as girls married and subsequently impregnated in adolescence are oftentimes both poor and undereducated. In light of the above, it appears intuitive that adolescent mothers would be at a higher risk of inadequate antenatal care. Moreover, further potential drivers can be responsible for inadequate ANC utilization by adolescents such as overall low agency of the adolescent mothers (what they are free to do and achieve in pursuit of goals or values they regard as important [11]) in the household or their perceived good health and hence lower prioritization for any kind of medical treatment. An ever-growing body of empirical literature appears to confirm this intuition; For example, Gogna and co-authors find that education plays a significant role in achieving the number of necessary ANC visits for adolescent mothers in Argentina. [12]. Ronen et al. conducted a nationally representative study in 120 maternal child health clinics in Kenya and report that the proportion of women attending at least 4 ANC visits was significantly lower in adolescents than in adults [13]. Similar findings were reported in Wakiso district in central Uganda [14] and in rural Burkina Faso [15]. Owolabi and collaborators analyse DHS data from 13 West African countries comparing ANC utilization of adolescent to adult mothers and find adolescents to be less likely to begin ANC within the first trimester and to adhere to at least 4 ANC visits [16].

With the present study we expand the existing literature by studying whether utilization of ANC services systematically differs between adolescent and non-adolescent mothers in the entire West and Central African region. We further investigate whether birth weight of adolescent mothers' children differs from birth weight of non-adolescent mothers’ children and to what extent these differences can be explained by adolescent mothers’ utilization of antenatal care services and socio-economic variables.

## METHODS

### Data sources

We pooled data from all available Demographic and Health Surveys (DHS) and Multi Indicator Cluster Surveys (MICS) from West and Central African countries. Countries in the sample include Benin, Burkina Faso, Cameroon, Central African Republic, Chad, Congo, Democratic Republic of Congo, Cote d'Ivoire, Gabon, Gambia, Ghana, Guinea, Guinea Bissau, Liberia, Mali, Mauritania, Niger, Nigeria, Senegal, Sierra Leone, and Togo and surveys were conducted between 1986 and 2017. The unit of observation are ever-married women in the age bracket of 15 to 49. We limited our analyses to women who have given birth at least once within their lifetime. Women who have not given birth yet but were pregnant at the time of the interview were excluded from our sample. The final sample included 963 751 women.

### Outcomes

Outcome variables are an indicator variable whether a woman received any antenatal care services during her last pregnancy and the number of ANC visits. As discussed above, while ANC4+ does no longer correspond to the officially recommended ANC schedule, this indicator is still commonly used as a benchmark indicator for minimum adequate ANC provision in resource-poor settings. We thus include an indicator variable for the presence of a skilled attendant at birth as well as an indicator variable that the (outdated) WHO recommendations of four ANC visits and the presence of a skilled attendant were met as additional outcome variables. Skilled attendant is defined as a doctor, midwife or auxiliary midwife, or other health worker (we also perform these analyses with more restrictive categories of doctor or midwife only). Further outcome variables are birth weight of the last-born child measured in grams as well as an indicator variable for low birth weight, which is defined as birth weight below 2500 g [17].

In the pooled sample information on ANC usage was available for 592 804 women and data on place of delivery for 482 012, with 479 187 having information on both variables of interest present. Information on child’s birth weight was available for 208 717 women.

### Exposure

The main exposure of interest is an indicator variable whether a mother was adolescent, defined as age 15-19 at birth of her last child. The original DHS sample includes some young adolescents below age 15 in individual surveys, however, as these children are not included in the sampling design of these surveys, we exclude them from our analyses. We controlled for household wealth measured by asset index quintiles, education of mother (categories: none or incomplete primary, primary or incomplete secondary, secondary or above), religion of the household head (categories: Muslim, Christian, Traditional, none or other) and primary sampling unit fixed effects.

### Statistical analysis

Descriptive statistics were calculated using survey weights for individual surveys. In pooled sample analyses, individual countries were additionally weighted according to their population share using World Bank Female Population Data [18]. Regressions were linear probability models for binary outcomes and linear regressions for discrete outcomes. Standard errors were clustered and fixed effects for primary sampling units (PSU) were included where specified. These fixed effects control for characteristics of the local environment that are shared by all people that live within the same PSU. Logistic regression or other nonlinear models can suffer from bias if a large number of fixed effects are included, therefore linear probability models are the preferred specification in this case. All analyses as well as sample generation have been performed using STATA 14 statistical software package (StataCorp, College Station, TX, USA).

## RESULTS

**Figure 1:**
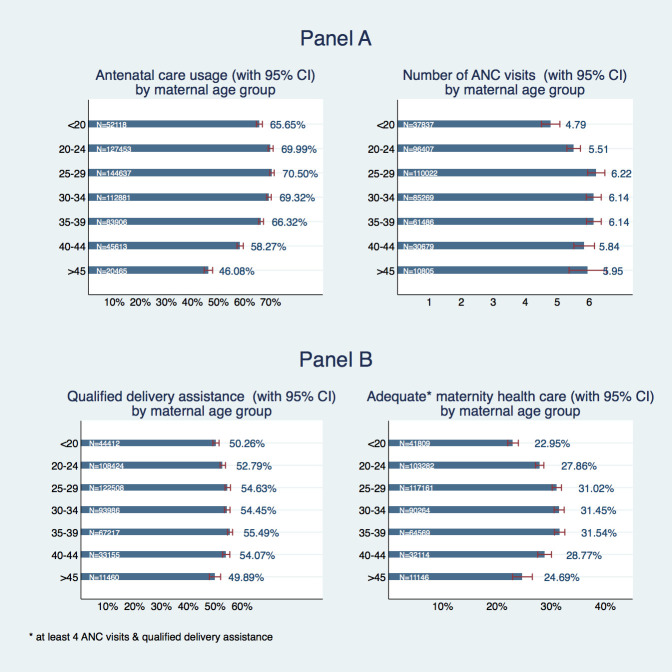
Utilization of antenatal care services by age of mother. **Panel A.** Any antenatal care (ANC) and number of ANC visits. **Panel B.** Qualified delivery assistance and appropriate maternal health care.

Panel A in **Figure 1** shows the proportion of mothers who received any sort of antenatal care and the average number of antenatal visits by age group. In younger and middle-aged mothers, adolescent mothers have the lowest share of ANC usage: 65.7% (95% confidence interval (CI) = 64.5%-66.8%) of adolescent mothers had at least one antenatal care visit compared to 70% (95% CI = 69%-70.9%) for women aged 20-24 years, 70.5% (95% CI = 69.7%-71.3%) for those aged 25-29 years and 69.32% (95% CI = 68.4%-70.2% for those aged 30-34 years. Only women above the age of 40 had an even lower ANC usage with 58.3% in age group 40-44 and 46.1% in age group of above 45 years of age. Conditional on having at least one ANC visit, adolescent mothers had on average 4.79 visits (95% CI = 4.50-5.07) and thus, on average, about 0.8 fewer visits than mothers in the neighboring age group 20-24 years (5.51 visits with 95% CI = 5.30-5.72) and 1.4 fewer visits than mothers aged 25-29 (6.22, 95% CI = 5.95-6.48), where the number of ANC visits peaked. All older age groups had a higher number of ANC visits with roughly 1-1.2 more visits, on average.

A skilled health care provider attended deliveries of adolescent mothers in 50.3% of cases (95% CI = 49.1%-51.4%), which is around 2.5 percentage points less than for the next higher age group with 52.8% of qualified delivery assistance (95% CI = 51.8%-53.8%) and 4-5 percentage points lower than age groups between 25 and 45 years of age. Only mothers above the age of 45 had an even slightly lower share of qualified delivery assistance with 49.9% (95% CI = 47.8%-52.0%) (**Figure 1**, Panel B). So, approximately 23% (95% CI = 22.0%-23.9%) of adolescent mothers received the minimum appropriate antenatal health care consisting of at least four ANC visits and skilled labor assistance. All remaining age groups had a higher share of appropriate maternal health care with 27.9% (95% CI = 27.1%-28.6%) in the 20-24 age group, roughly 31% at ages 25-39 and 24.7% (95% CI = 22.9%-26.5%) in the oldest age group.

To investigate to what extent these differences in ANC usage of adolescent vs non-adolescent mothers can be explained by differences in their socio-economic status, we estimate various regression models presented in **Table 1**. Columns (1), (3), (5) and (7) show results from simple regression models with an indicator variable for adolescent mother as the only explanatory variable and the four dependent variables ANC usage (indicator), number of ANC visits (discrete), indicator variable for qualified delivery attendance, and an indicator variable for appropriate minimum level of antenatal care as outcomes. The coefficients basically reflect the differences between adolescent and non-adolescent mothers that can also be observed in the descriptive statistics, without any additional controls. Columns (2), (4), (6), and (8) show the results of multiple regressions of the same exposure and outcome variables, in addition controlling for household wealth, education of the mother, household religion, and primary sampling unit fixed effects (PSU FE). All coefficients of the indicator variable for adolescent mother in the multiple models remained statistically significant. So even when confounding factors such as wealth, education, or local environment were controlled for, the difference in adolescent and adult outcomes was statistically significantly different from zero: adolescents had, on average, 0.35 fewer ANC visits, a roughly 2 percentage points lower probability of skilled delivery assistance, and a 4 percentage points lower probability of receiving appropriate minimum maternal health care, and a 1 percentage point lower probability of receiving any antenatal care. Moreover, wealth and education were statistically significantly positively associated with all outcomes of interest: eg, belonging to the richest wealth quintile (top 20% of asset wealth) was associated with 2.8 more ANC visits and a 22.4 percentage point higher probability of receiving appropriate minimum maternal health care as compared to the asset-poorest 20% of the population, all other factors being equal. Having secondary or higher education had an even stronger association with all outcomes with 3.6 more ANC visits and a 27.6 percentage point higher probability of receiving adequate health care in pregnancy. Interestingly, differences in outcomes between various religious groups were also statistically significantly different from zero with fairly high point estimates and a consistent direction: belonging to Christian or no religion had a positive association with all indicators compared to Muslim religion. Belonging to traditional religion, in turn, had a negative association. In other words, all other factors being equal, women belonging to traditional religion would be predicted to do worst, and women belonging to Christian religion best on all four indicators for antenatal care usage.

**Table 1:** Results of various regression models (outcome variable as column heading)*

	Antenatal care (dummy), LPM		Nr. of ANC visits OLS			Skilled delivery assistance, LPM		Adequate maternity health care, LPM			
	**Simple**	**Multiple**		**Simple**	**Multiple**		**Simple**	**Multiple**	**Simple**	**Multiple**	
		**PSU FE**			**PSU FE**			**PSU FE**		**PSU FE**	
Adolescent	0.017‡ (0.002)	-0.008‡ (0.003)		-0.637‡ (0.073)	-0.349‡ (0.098)		-0.019‡ (0.002)	-0.018‡ (0.003)	-0.044‡ (0.002)	-0.039‡ (0.003)	
Wealth quintile:											
Second quintile		0.049‡ (0.004)			0.656‡ (0.096)			0.044‡ (0.004)		0.041‡ (0.003)	
Middle quintile		0.087‡ (0.005)			1.249‡ (0.107)			0.081‡ (0.005)		0.090‡ (0.004)	
Fourth quintile		0.109‡ (0.005)			1.936‡ (0.123)			0.120‡ (0.005)		0.147‡ (0.004)	
Richest quintile		0.134‡ (0.006)			2.767‡ (0.154)			0.160‡ (0.007)		0.224‡ (0.005)	
Maternal education:											
Primary or incomplete secondary		0.055‡ (0.002)			0.586‡ (0.091)			0.105‡ (0.003)		0.164‡ (0.003)	
Secondary or higher		0.077‡ (0.004)			3.632‡ (0.232)			0.152‡ (0.005)		0.276‡ (0.006)	
Religion (ref.: Muslim):											
Christian		0.059‡ (0.003)			0.809‡ (0.101)			0.007† (0.004)		0.070‡ (0.003)	
Traditional		-0.028‡ (0.008)			-0.855‡ (0.154)			-0.132‡ (0.008)		-0.034‡ (0.006)	
None or other		0.044‡ (0.006)			0.026‡ (0.200)			0.001 (0.007)		0.052‡ (0.006)		
Number of observations	592 840	366 443		432 903	286 127		482 012	300 731	460 959	291 564	

OLS – ordinary least squares, LPM – linear probability model, PSU FE – primary sampling unit fixed effects

*Standard errors in parentheses.

†*P* < 0.05.

‡*P* < 0.001.

**Figure 2:**
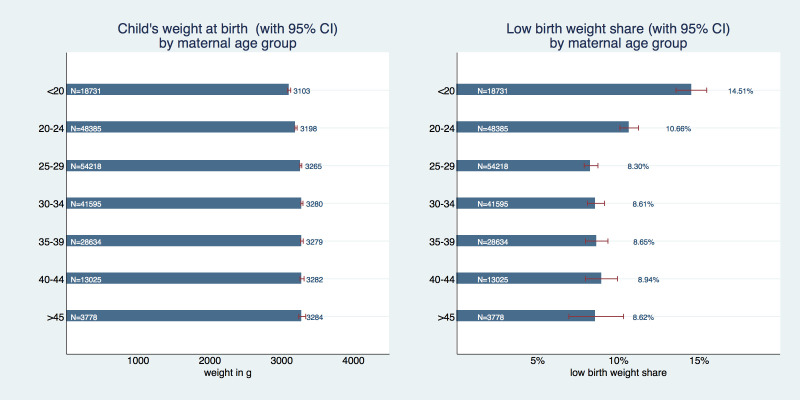
Child’s birth weight in grams and low birth weight share by age of mother.

Children of adolescent mothers had a lower birth weight (**Figure 2**) of on average 3103 g (95% CI = 3082-3124g) compared to almost 100g more – 3198 g (95% CI = 3183-3214 g) – in the 20-24 age group, 3265 g (95% CI = 3251-3278 g) in the 25-29 age group, 3280 g (95% CI = 3266-3294 g) in the 30-34 age group, 3280 g (95% CI = 3260-3299 g) in the 35-39 age group, 3282 g (95% CI = 3254-3311 g) in the 40-44 age group, and 3284 g (95% CI = 3235-3333 g) in the 45+ age group. This results in a share of 14.5% (95% CI = 13.6%-15.5%) of low-birth weight among adolescent mothers’ infants, which is almost 1.5 times higher than in the 20-24 age group with 10.7% (95% CI = 10.1%-11.2%) and almost two times higher than for all other age groups (shares between 8.3% and 8.9%).

To investigate to what extent these differences in infantile birth weight and probability of low birth weight-delivery can be explained by adolescent women's socio-economic characteristics and their lower propensity to use ANC services, we estimated various regression models shown in **Table 2** for infantile birth weight in kilograms (kg) and **Table 3** for a dummy variable of low-birth weight. Column (1) shows the results of a simple model with birth weight/low-birth weight indicator as outcome and an indicator for adolescent mother as exposure. Column (2) shows the multiple regression results with the same outcomes and exposure, in addition controlling for household wealth, mother's education, household religion, and primary sampling unit fixed effects. Columns (3) and (4) repeat these regressions adding a control variable for any ANC visits (indicator), columns (5) and (6) instead add a discrete control variable for the number of ANC visits, and columns (7) and (8) add a control variable for having received adequate minimum maternal health care (at least four ANC visits during pregnancy as well as skilled labor assistance). The indicator variable for adolescent mother is statistically significant in all specifications and the magnitude of the coefficients only modestly changes between the different specifications. Tables S1 and S2 in the **Online Supplementary Document** redo the same regressions adding maternal BMI to the control variables. Despite the importance of mother's BMI, this was not chosen as main specification because the inclusion of mother's BMI reduces the sample by more than half due to missing observations. The general patterns however remain the same, the coefficients for adolescent mother are all statistically significant and differences in magnitudes between the specifications are rather modest.

**Table 2:** Outcomes of simple and multiple regression models with PSU FE (outcome variable: last-born child’s weight at birth in kg)*

	(1)	(2)	(3)	(4)	(5)	(6)	(7)	(8)
	**Simple model**	**Multiple model with SES**	**Simple model (1) + ANC**	**Multiple model (2) + ANC**	**Simple model (1) + nr. ANC visits**	**Multiple model (2) + nr. ANC visits**	**Simple model (1) + adequate mat. HC**	**Multiple model (2) + adequate mat. HC**
Adolescent	-0.136‡ (-23.61)	-0.119‡ (-16.21)	-0.136‡ (-24.02)	-0.119‡ (-16.27)	-0.130‡ (-21.36)	-0.117‡ (-14.84)	-0.129‡ (-21.43)	-0.115‡ (-14.93)
Received antenatal care			-0.029† (-2.70)	-0.028 (-1.61)				
Times received ANC					0.002‡ (11.48)	0.001‡ (6.22)		
Adequate maternal HC							0.081‡ (23.96)	0.058‡ (13.06)
Wealth quintile:								
Second		-0.011 (-1.47)		-0.010 (-1.43)		-0.013 (-1.69)		-0.013 (-1.77)
Middle		-0.009 (-1.26)		-0.009 (-1.22)		-0.009 (-1.21)		-0.011 (-1.39)
Fourth		0.001 (0.08)		-0.000 (-0.00)		0.002 (0.26)		-0.002 (-0.25)
Richest		0.006 (0.72)		0.005 (0.64)		0.004 (0.48)		-0.001 (-0.12)
Maternal education:								
Primary or incomplete secondary		0.046‡ (9.34)		0.046‡ (9.25)		0.047‡ (9.36)		0.038‡ (7.42)
Secondary or higher		0.053‡ (5.66)		0.051‡ (5.44)		0.049‡ (5.12)		0.034‡ (3.45)
Religion (ref.: Muslim):								
Christian		0.100‡ (18.29)		0.100‡ (18.16)		0.095‡ (16.83)		0.100‡ (17.54)
Traditional		-0.015 (-1.25)		-0.015 (-1.26)		-0.022 (-1.77)		-0.010 (-0.76)
None or other		0.054‡ (5.31)		0.053‡ (5.20)		0.049‡ (4.76)		0.052‡ (4.91)
Observations	208 717	141 788	207 283	140 600	188 168	132 882	189 545	132 937

SES – socio-economic status, ANC – antenatal care, HC – health care

*t statistics in parentheses.

†*P* < 0.01.

‡*P* < 0.001.

**Table 3:** Outcomes of simple and multiple regression models with PSU FE (outcome variable: low birth weight of last-born child)*

	(1)	(2)	(3)	(4)	(5)	(6)	(7)	(8)
	**Simple model**	**Multiple model with SES**	**Simple model (1) + ANC**	**Multiple model (2) + ANC**	**Simple model (1) + nr. ANC visits**	**Multiple model (2) + nr. ANC visits**	**Simple model (1) + adequate mat. HC**	**Multiple model (2) + adequate mat. HC**
Adolescent	0.0544§ (20.83)	0.0489§ (14.63)	0.0541§ (22.44)	0.0490§ (14.65)	0.0504§ (19.50)	0.0465§ (13.23)	0.0515§ (20.17)	0.0469§ (13.33)
Received antenatal care			-0.0391§ (-8.54)	-0.0399§ (-5.98)				
Times received ANC					-0.0003§ (-5.82)	-0.0002‡ (-3.02)		
Adequate maternal HC							-0.0229§ (-15.94)	-0.0130§ (-7.45)
*Wealth quintile:*								
Second		-0.00323 (-1.09)		-0.00312 (-1.05)		-0.00322 (-1.09)		-0.00267 (-0.92)
Middle		-0.009‡ (-3.13)		-0.0087‡ (-3.04)		-0.0081‡ (-2.84)		-0.0093‡ (-3.26)
Fourth		-0.0108§ (-3.68)		-0.0103§ (-3.50)		-0.0102§ (-3.50)		-0.0108§ (-3.68)
Richest		-0.0118§ (-3.81)		-0.0112§ (-3.59)		-0.00988‡ (-3.12)		-0.0105§ (-3.37)
*Maternal education:*								
Primary or incomplete secondary		-0.0079§ (-4.02)		-0.0075§ (-3.84)		-0.0076§ (-3.78)		-0.0054‡ (-2.61)
Secondary or higher		-0.0111‡ (-3.28)		-0.0109‡ (-3.16)		-0.0102‡ (-2.95)		-0.0072† (-2.07)
*Religion (ref.: Muslim):*								
Christian		-0.0240§ (-11.41)		-0.0246§ (-11.64)		-0.0233§ (-10.94)		-0.0244§ (-11.40)
Traditional		-0.0195§ (-3.95)		-0.0197§ (-3.99)		-0.0200§ (-3.78)		-0.0228§ (-4.34)
None or other		-0.0177§ (-4.19)		-0.0184§ (-4.33)		-0.0154§ (-3.59)		-0.0149§ (-3.46)
Observations	208 717	141 788	207 283	140 600	188 168	132 882	189 545	132 937

PSU FE – primary sampling unit fixed effects SES – socio-economic status, ANC – antenatal care, HC – health care

*t statistics in parentheses.

†*P* < 0.05.

‡*P* < 0.01.

§*P* < 0.001.

## DISCUSSION

We have documented that adolescent mothers in West and Central Africa have a systematically lower utilization of antenatal care services than non-adolescent mothers and their children on average have lower birth weight and consequently a higher probability of low birth weight. The lower utilization of antenatal care services is still visible in a regression controlling for a range of socio-economic characteristics, including local environment of the women. The same is true for birth weight and the probability of low birth weight suggesting that socio-economic characteristics and utilization of antenatal care services do not explain the difference in birth weight outcomes between adolescent and non-adolescent mothers. This implies that other individual level factors, which remain unobserved in the underlying data, must explain this difference. Possible individual-level determinants of low birth weight might include unhealthy behaviors during pregnancy such as inadequate diets or excessive physical strain or physiological factors, such as overall physical fitness of the young organism for gestation.

In general, our estimates should be interpreted as lower bounds of the true differences between adolescent and adult mothers. For one, we exclude pregnant women from these analyses, as we cannot reliably adjust for gestational age to assess the appropriate number of ANC visits. When including these data points, the differences are more pronounced. Moreover, we excluded a substantial number of observations where data was somewhat inconsistent as some of the variables we used in the construction of our outcome indicators were missing but could, generally speaking, be imputed from other variables. Including these data points also increased the difference between adolescent and adult age groups; however, we decided to exclude it from main analyses in favor of a more transparent variable coding.

Another aspect that might confound our analysis is parity (the number of children born to a woman): empirical literature generally confirms that ANC and institutional delivery utilization decrease with parity [19,20]. Limiting our analyses to primapari (women who gave birth for the first time in their life, results not presented), average ANC usage and institutional delivery rates increase for all age groups but more so for the adult subgroups than for adolescents (as, naturally, there are more higher-order births in older age groups so this “routine” may bias our estimates of adult ANC utilization downwards). In the primapari subset, we find the ANC usage to increase within the magnitude of about one percentage point for the youngest age group but of up to ten percentage points for higher-age groups. However, such a restriction dwindles the sample size dramatically rendering sensible regression analysis impossible.

In the presented analyses we rely on a specific number of four ANC visits as a quality criterion for adequate maternal health care. We discussed the historical development of this indicator in the introduction section and are aware of the drawbacks of such an indicator (beyond ANC4+ being no longer recommended), as it cannot control for heterogeneity of treatment. One interesting approach to circumventing this problem is attempting to use information on treatments received by pregnant women within their ANC visits rather than their sheer number. Hodgins and D’Agostino suggest a number of interventions recorded by the DHS that can be considered good practice in antenatal care and can serve as a proxy for its overall quality. These include blood pressure measurement, tetanus toxoid vaccination, urine testing, iron-folate supplementation, etc. [6]. Unfortunately, this information is only available for a small subset of women in our dataset which does not allow for reliable estimates.

Lastly, our study design does not allow making any claims of causality and the results of this study have to be interpreted as descriptive.

## CONCLUSIONS

Our findings suggest that adolescent mothers in West and Central Africa are at a higher risk of receiving inadequate antenatal care and subsequently giving birth to low birth weight infants, compared to adult mothers. Both exposures (being an adolescent and lower ANC usage) are independently associated with low birth weight outcomes suggesting that there are unobserved characteristics of adolescents that correlate with low infantile birth weight. Hence it is necessary to specifically tailor ANC supply to adolescents’ needs on the one hand, and to promote information on ANC benefits on the other, and to stress their exigency in context of adolescent pregnancy in adolescent mothers’ immediate surroundings – husbands, in-laws, and parents. Increasing pregnant adolescent girls’ agency within their families might also positively affect their health care utilization, which could, for instance, be promoted with conditional cash transfers to particularly vulnerable young girls directly. Conditional cash transfers have been empirically shown to have the potential to improve ANC utilization in low-resource settings [21-23]. In addition, conditional cash transfers targeting adolescent girls have been shown to increase the girls’ empowerment and to improve their nutritional, health, and schooling outcomes [24]. Targeting pregnant adolescent girls with carefully designed interventions might potentially have the power to achieve both of the above. Moreover, it is also necessary to strengthen the supply side by expanding ANC services for adolescents, who are naturally less knowledgeable about maternal care, with informational interventions to ensure that young girls know what is in their right to expect from ANC providers to empower them to demand adequate, timely, and goal-oriented services.

Lastly, household survey programs such as DHS and MICS would substantially improve the information base pertaining to questions of adolescent maternal health by developing a set of survey questions which aim at measuring ANC service quality rather than its mere frequency and advocate for their routine inclusion in standard questionnaires in every participating country.

## Additional material


Online Supplementary Document
