# Supplementary material for: Does early childbearing affect utilization of antenatal care services and infant birth weight: Evidence from West and Central African Region
Source: J Glob Health. 2021 Aug 10;11:13003. doi: 10.7189/jogh.11.13003 (PMC8397281; doi:10.7189/jogh.11.13003)
Supplement: Online Supplementary Document [file jogh-11-13003-s001.pdf]

Table S1: Outcomes of simple and multiple regression models with PSU FE, Outcome variable: Last child's weight at birth in kg

	(1)	(2)	(3)	(4)	(5)	(6)	(7)	(8)
	Simple model	Multiple model with SES+BMI	Simple model (1) + ANC	Multiple model (2) + ANC	Simple model (1) + nr. ANC visits	Multiple model (2) + nr. ANC visits	Simple model (1) + adequate mat. HC	Multiple model (2) + adequate mat. HC
Adolescent	-0.136***	-0.0775***	-0.136***	-0.0781***	-0.130***	-0.0774***	-0.129***	-0.0751***
	(-23.61)	(-6.12)	(-24.02)	(-6.15)	(-21.36)	(-6.13)	(-21.43)	(-5.84)
Maternal BMI		0.0158***		0.0158***		0.0157***		0.0155***
		(19.52)		(19.27)		(19.45)		(18.97)
Received antenatal care			-0.0290**	-0.0271				
			(-2.70)	(-1.02)				
Times received ANC					0.002***	0.0007**		
					(11.48)	(3.16)		
Adequate maternal HC							0.0813***	0.0588***
Wealth quintile:							(23.96)	(9.09)
second		-0.0153		-0.0150		-0.0155		-0.0171
		(-1.42)		(-1.39)		(-1.45)		(-1.57)
middle		-0.0195		-0.0194		-0.0198		-0.0204
		(-1.85)		(-1.82)		(-1.86)		(-1.89)
fourth		-0.0213*		-0.0221*		-0.0218*		-0.0246*
		(-1.99)		(-2.06)		(-2.04)		(-2.25)
richest		-0.0464***		-0.0472***		-0.0471***		-0.0505***
		(-3.90)		(-3.94)		(-3.96)		(-4.17)
Maternal education:								
primary or incomplete secondary		0.0637***		0.0635***		0.0635***		0.0545***
		(8.53)		(8.44)		(8.50)		(7.05)
secondary or higher		0.0840***		0.0829***		0.0812***		0.0641***
		(6.25)		(6.08)		(6.03)		(4.65)
Religion (ref.: Muslim)								
Christian		0.0600***		0.0604***		0.0602***		0.0617***
		(7.67)		(7.60)		(7.68)		(7.73)
Traditional		-0.0533**		-0.0537**		-0.0524**		-0.0445**
		(-3.18)		(-3.21)		(-3.12)		(-2.65)
none or other		0.0057		0.0045		0.0062		0.0076
		(0.44)		(0.34)		(0.48)		(0.57)
Constant	3.193***	2.811***	3.220***	2.837***	3.189***	2.808***	3.157***	2.790***
	(1596.47)	(140.58)	(302.76)	(88.68)	(1607.53)	(140.28)	(1301.30)	(139.22)
Observations	208717	63700	207283	62780	188168	63660	189545	61627

t-statisctics in parentheses
* *p < 0.05,* ** *p < 0.01,* *** *p < 0.001*

Table S.2: Outcomes of simple and multiple regression models with PSU FE, Outcome variable: Low birth weight of last child

	(1)	(2)	(3)	(4)	(5)	(6)	(7)	(8)
	Simple model	Multiple model with SES+BMI	Simple model (1) + ANC	Multiple model (2) + ANC	Simple model (1) + nr. ANC visits	Multiple model (2) + nr. ANC visits	Simple model (1) + adequate mat. HC	Multiple model (2) + adequate mat. HC
Adolescent	0.0544***	0.0419***	0.0541***	0.0423***	0.0504***	0.0418***	0.0515***	0.0420***
	(20.83)	(7.62)	(22.44)	(7.68)	(19.50)	(7.60)	(20.17)	(7.58)
Maternal BMI		-0.0034***		-0.0034***		-0.0034***		-0.0035***
		(-10.65)		(-10.51)		(-10.60)		(-10.72)
Received antenatal care			-0.0391***	-0.0446***				
			(-8.54)	(-4.56)				
Times received ANC					-0.0003***	-0.0002*		
					(-5.82)	(-2.12)		
Adequate maternal HC							-0.0229***	-0.0107***
							(-15.94)	(-4.12)
Wealth quintile:								
second		-0.0064		-0.0063		-0.0064		-0.0059
		(-1.49)		(-1.46)		(-1.50)		(-1.37)
middle		-0.0069		-0.0067		-0.0068		-0.0069
		(-1.54)		(-1.51)		(-1.53)		(-1.51)
fourth		-0.0057		-0.0053		-0.0057		-0.006
		(-1.29)		(-1.18)		(-1.27)		(-1.31)
richest		-0.0011		-0.0001		-0.001		-0.0002
		(-0.22)		(-0.03)		(-0.20)		(-0.05)
Maternal education:								
primary or incomplete secondary		-0.0104***		-0.0104***		-0.0103***		-0.00796**
		(-3.48)		(-3.50)		(-3.48)		(-2.60)
secondary or higher		-0.0132**		-0.0129**		-0.0123**		-0.00994*
		(-2.83)		(-2.72)		(-2.64)		(-2.06)
Religion (ref.: Muslim)								
Christian		-0.0226***		-0.0228***		-0.0227***		-0.0231***
		(-7.05)		(-7.09)		(-7.06)		(-7.07)
Traditional		-0.0176*		-0.0179*		-0.0178*		-0.0204**
		(-2.32)		(-2.35)		(-2.35)		(-2.62)
none or other		-0.0163**		-0.0167**		-0.0165**		-0.0154**
		(-2.83)		(-2.89)		(-2.86)		(-2.62)
Constant	0.107***	0.203***	0.145***	0.246***	0.107***	0.204***	0.117***	0.209***
	(139.39)	(25.01)	(31.96)	(19.93)	(127.57)	(24.98)	(113.78)	(25.82)
Observations	208717	63700	207283	62780	188168	63660	189545	61627

t-statisctics in parentheses

* *p < 0.05*, ** *p < 0.01*, *** *p < 0.001*
